# Easy triplex: An online tool for predicting the formation of DNA—RNA triple helices

**DOI:** 10.1016/j.csbj.2025.04.008

**Published:** 2025-04-09

**Authors:** Lintao Xian, Jianming Liu, Shunshun Zhu, Fan Liang, Hao Luo, Li Zhao, Min Shi, Cheng Gong, Zhen Li, Tao Guo

**Affiliations:** a Department of Intelligent Medical Engineering, School of Basic Medical Sciences, Shandong Second Medical University, Weifang 261053, China; b Department of Pathophysiology, School of Basic Medical Sciences, Shandong Second Medical University, Weifang 261053, China; c Department of Biochemistry and Molecular Biology, School of Basic Medical Sciences, Shandong Second Medical University, Weifang 261053, China; d Department of Pathology, School of Basic Medical Sciences, Shandong Second Medical University, Weifang 261053, China; e Department of Clinical Microbiology, School of Laboratory Medicine, Shandong Second Medical University, Weifang 261053, China; f Department of Hepatobiliary and Pancreatic Surgery, Zhongnan Hospital of Wuhan University, Wuhan 430071, China

**Keywords:** Online tool, Non-coding RNA, Hoogsteen pairing, Triplex

## Abstract

Increasing evidence supports the idea that intranuclear noncoding RNAs interact with target DNA to regulate their expression. The structural basis of this process involves the formation of DNA—RNA triple helices without unwinding the DNA double helix. This triplex formation process relies on the base pairing of the triplex target site (TTS) and the triplex-forming oligonucleotide (TFO), making the prediction of their binding crucial for mechanistic investigations. Here, we propose an online tool called Easy Triplex for predicting the formation of DNA—RNA triplexes. Using the canonical Hoogsteen pairing rules, Easy Triplex matches and predicts the details and distribution of all TTSs and TFOs on the basis of the user-provided DNA and RNA sequences. Practical application has shown that Easy Triplex can efficiently and accurately predict known triplex information. Its flexible parameter settings and scientifically set default output thresholds make Easy Triplex suitable for predicting the TFOs of both long and short noncoding RNAs. Additionally, users can choose to export the distribution information of CpG islands on the DNA sequence for further TTS screening. Thus, Easy Triplex is a simple and convenient online tool for predicting triplex formation. Easy Triplex is publicly accessible at http://easy-triplex.com/.

## 1 Introduction

Since the last century, it has been known that DNA and RNA can form a triple helix structure known as a triplex, which plays a crucial role in DNA damage repair, RNA initiation, metabolism, and transcriptional regulation processes [1], [2]. As the structural foundation for DNA—RNA interactions, the triplex can act without unwinding the DNA double helix, relying solely on RNA—DNA base pairing. This simple and energy-efficient mode of regulation can significantly impact the expression of target genes [3]. This regulatory mechanism primarily involves RNA as an upstream regulatory molecule that binds to specific complementary regions of DNA, thereby affecting the expression of downstream or neighboring genes. The RNA segment that forms the triple helix structure is referred to as the triplex-forming oligonucleotide (TFO), whereas the double-stranded DNA sequence is referred to as the triplex target site (TTS). Therefore, the triplex can be viewed as a structure formed by the binding of TFO and TTS [4].

**Fig. 1:**
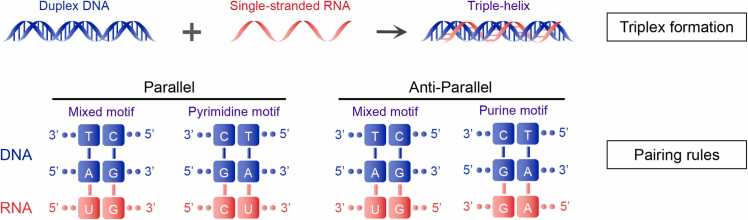
Diagram depicting the formation of a triplex structure between RNA and DNA, illustrating adherence to the canonical Hoogsteen pairing rules and resulting in four distinct motifs.

In the formation of the triplex structure, the duplex DNA adheres to Watson—Crick base pairing, maintaining the integrity of the double-stranded structure, whereas the third RNA strand is accommodated in the major groove of the DNA. The third strand associates with duplex DNA by establishing Hoogsteen or reverse Hoogsteen hydrogen bonds with a purine-rich DNA strand, adopting either a parallel or an anti-parallel orientation [5]. With respect to base pairing rules, the RNA bases involved in matching can be classified as purine bases, pyrimidine bases, and mixed bases. Parallel base pairing primarily consists of pyrimidine motifs (C and U) and mixed motifs (U and G), forming three types of pairings, including TA:U, CG:G, and CG:C [6]. Anti-parallel pairing mainly consists of a purine motif (G and A) and a mixed motif (U and G), forming 3 types of pairings, including TA:U, CG:G, and TA:A [7]. In summary, the triplex structure encompasses 4 canonical rules of DNA—RNA pairing (Fig. 1).

In recent years, studies on non-coding RNAs have increasingly highlighted their potential as upstream regulatory molecules, capable of modulating the expression and function of target genes [8]. Nuclear noncoding RNAs, particularly long noncoding RNAs (lncRNAs), directly interact with DNA and regulate genes associated with target DNA [9], [10]. The structural basis for this regulation largely involves triplex structures characterized by Hoogsteen pairing [11]. The validation of this TFO-TTS structure is therefore an essential component of current molecular mechanism research. Hence, predictions regarding the binding potential between RNA and DNA sequences play a crucial role.

Our team recently developed an online prediction tool called Easy Triplex. Users can input sequences of RNA and target DNA they wish to investigate, and the tool then performs matching analysis based on the aforementioned Hoogsteen base pairing. The output results depend on the parameters set by the user, including the number of consecutive pairing bases in the triplex and the error rate. These parameters can be customized according to the user's research needs. Multiple output results are arranged according to the length of the triplex, and detailed information for each entry is available for review. Additionally, Easy Triplex performs CpG island prediction on the basis of the user-input DNA sequence and can choose to display it with the TTS according to the user's preference. Therefore, this online tool will provide researchers with convenient assistance in investigating DNA-RNA interactions. Easy Triplex is now available at http://easy-triplex.com/.

## 2 Methods

### 2.1 Pairing Rules and Parameter Set

Our computational tool operates based on 4 canonical coding rules, which include different orientations (Fig. 1). The required input parameters include the minimum number of consecutive pairs essential for triplex formation and the maximum allowable error rate. A "pair" is defined as any set of three nucleotides that conforms to the canonical code. The "error rate" is defined as the proportion of DNA—RNA matches within a certain length of triplex that do not adhere to the aforementioned pairing rules. To facilitate the prediction of TFOs for both long-chain RNA (such as lncRNA) and short-chain RNA (e.g., nuclear activating micro RNA), a minimum contiguous pair requirement of seven or more has been established, with a maximum allowable error rate set at 20 %. Notably, even in cases where the mismatch rate is not zero, it is limited to consecutive sequences of A and G. For the predicted TTS, the presence of any C or T is not allowed. Consequently, users can customize the matching thresholds according to their specific needs and anticipated cellular processing conditions.

### 2.2 Pairing Operations

**Fig. 2:**
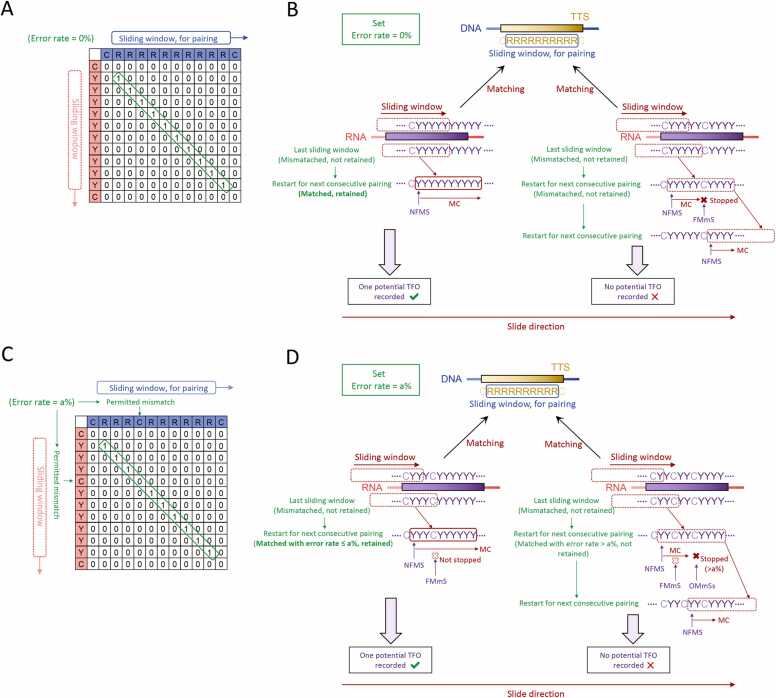
**Sliding window operation under different error rates.** When the error rate is set to zero, (A) the algorithmic rules of the sliding window in a specific frame and (B) the schematic diagram of the window sliding process. When the mismatch rate is nonzero, (C) the algorithmic rules of the sliding window in a specific frame and (D) the schematic diagram of the window sliding process. All windows are before expansion and a> 0. In Panel 2 A and 2 C, 0 and 1 respectively represent mismatches and matches, and the green box indicates a sequence within the two sliding windows that exhibits consecutive matches meeting the established threshold. MC, matching computation; NFMS, new first matching site; FMmS, first mismatching site; OMmSs, other mismatching sites.

Building upon the aforementioned pairing rules, Easy Triplex requires the execution of processes and computations related to multiple sequence alignment. This tool performs sliding window operations on user-provided DNA and RNA sequences, employing an algorithm inspired by the latest advancements in sliding window methodology, with subtle modifications to the output data [12]. The DNA input field exclusively recognizes the nucleotides represented by the letters A, G, C, and T, whereas the RNA input field recognizes A, G, C, T and U. In accordance with the 4 Hoogsteen canonical codes, the 2 input sequences are subjected to matching operations across 4 distinct frames. Within each frame's specific regulatory context, the first matching nucleotide facilitates a sliding operation toward the corresponding nucleotide in both input sequences, allowing for continuous matching and temporary memory retention. The sliding window can be conceptualized as a confined, cyclic internal memory reliant on an array structure [13]. Data transition sequentially according to a specified step size within the array, whereby older data are displaced to accommodate new entries into the window. Consequently, the window size remains constant throughout the execution of a specific matching operation. When the mismatch rate is set to zero, the first mismatch within the sliding window will be detected, causing the matching process to restart from the position immediately following the last mismatch (Fig. 2A-B, Supplementary Figure S1A). When the mismatch rate is set to a nonzero value, the occurrence of the first mismatch in the sliding window does not immediately terminate the ongoing computation or discard temporary data, unless the total mismatch rate within the window exceeds the predefined error threshold (Fig. 2C-D, Supplementary Figure S1B). Rather, it prompts a dual-layered iterative process. After the parameters of the sliding match window are established, the window is first fixed, followed by expansion, before the sliding match is reinitiated to ensure that subsequent continuous matching operations are maintained.

### 2.3 Prediction of CpG islands (optional)

Easy Triplex additionally offers the ability to predict and display the locations of CpG islands within a given DNA sequence. On the basis of the characterization of CpG islands in DNA sequences (greater than 200 bp in length, over 50 % GC content, and a ratio of observed to expected CpG sites greater than 0.6) [14], we adopted the computational prediction methodology utilized by the UCSC Genome Browser [15]. This approach assigns scores to the nucleotides G and C and determines the relative position of CpG islands based on these scores using a continuous stacking computation. Rather than utilizing a sliding-window search for identifying CpG islands, this computation implements a running-sum scoring system, wherein the presence of a 'C' followed by a 'G' increases the score by 17. In contrast, any other combination decreases the score by 1. When the score transitions from a positive value to zero (or at the end of the sequence), the current sequence span is assessed to determine if it qualifies as a CpG island. The search process subsequently recurs from the position of the maximum running score to the current position. Easy Triplex executes the corresponding computations on the basis of the user-provided sequences, ultimately displaying the predicted spans and locations of the identified CpG islands in the results.

### 2.4 Triplex Optimization and Filtering in Specific Situations

**Fig. 3:**
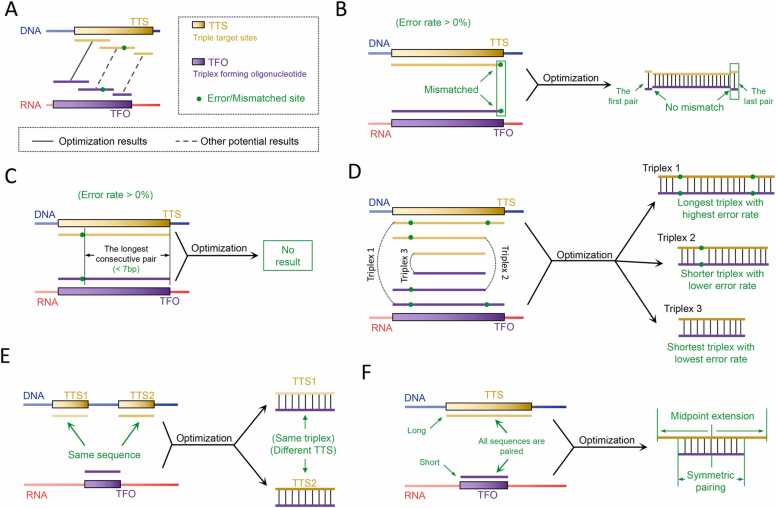
**Illustration of the Optimal Algorithm Principles.** (A) Schematic of overall screening principles; (B) The first and last bases of the triplex are supposed to be paired; (C) Valid triplexes must ensure a minimum of 7 consecutive base matches; (D) Only the longest triplex is reserved among those with equal error rates, while shorter triplexes with lower error rates are also retained; (E) Triplexes with identical sequences may be formed by different TTSs or TFOs, and their results should all be preserved; (F) Only triplexes with midpoint matches are retained for sequences with continuous sliding pairing.

The optimization and filtering process focuses primarily on the length of triplex structures and the rate of mismatches, aiming to ensure that the triplexes are both longer and exhibit a lower mismatch rate. Consequently, within the same pairing region, triplexes characterized by shorter lengths or higher mismatch rates are ignored in the computational results (Fig. 3A). The following criteria are applied for filtering in several specific scenarios:

1) For calculations where the error rate is nonzero, the exported triplexes must have both the first and last bases correctly matched (Fig. 3B, Supplementary Figure S2A).
2) Among all the valid results, irrespective of the pairing frame employed, each triplex must contain at least seven consecutive base pairs without mismatches (Fig. 3C, Supplementary Figure S2B).
3) Among the triplexes sharing the same error rate within the same TTS/TFO region, only the longest triplex is retained. However, if the error rate decreases or becomes zero, the corresponding shorter triplexes remain valid results and are retained (Fig. 3D, Supplementary Figure S2C).
4) If two or more identical TFOs or TTSs appear simultaneously in either the RNA or DNA sequences, the corresponding triplex information for all instances are retained and deemed valid (Fig. 3E, Supplementary Figure S2D).
5) In instances of continuous sliding matches for the same sequences, a unique match is determined based on the midpoint of the longer sequence between the TFO and TTS. If both the TFO and TTS consist of odd or even bases, matching occurs at one or two corresponding bases positioned centrally. Conversely, if one of the sequences is odd and the other is even, matching involves the central base from the odd sequence and the two central bases from the even sequence, simultaneously performing two sliding matches, with both results retained (Fig. 3F, Supplementary Figure S2E).

## 3 Results

### 3.1 Standard Workflow

**Fig. 4:**
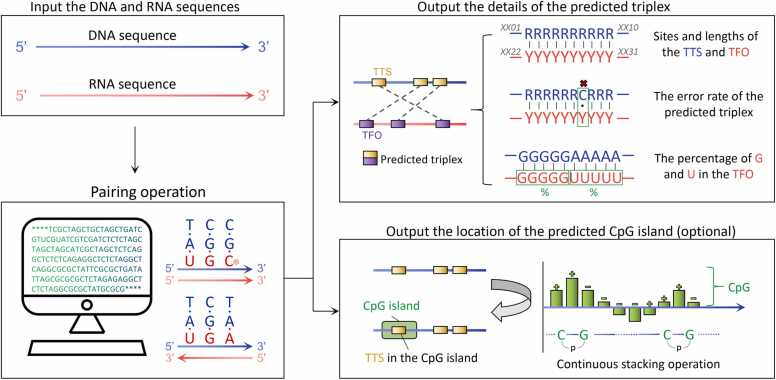
**Workflow of Easy Triplex.** After the input of DNA and RNA sequences, our tool conducts online computations on the basis of Hoogsteen pairing rules. The output presents a detailed listing of all triplexes and their relevant information. Users also have the option to choose whether to display information regarding CpG islands on the DNA sequence according to their preferences.

Upon logging into our website, users can select the "Online Tool" section and input the desired DNA and RNA sequences in the 5′ to 3′ orientation. Following the completion of input, the website conducts result computations and validity assessments on the basis of the aforementioned matching rules and algorithms. Once the computation is complete, the displayed results include detailed information about the predicted triplexes, encompassing the positions and lengths of the TTS and TFO, the error rate within the predicted triplexes, and the percentage of G and U within the TFO. Additionally, users have the option to choose whether to display information related to CpG islands based on their requirements. If the resulting information for the CpG islands is activated, the relative positions of the CpG islands are displayed alongside the location information of the predicted TTS, providing users with the convenience of filtering the TTS information related to the CpG islands and their respective TFOs (Fig. 4).

### 3.2 Input and Output Presentations

**Fig. 5:**
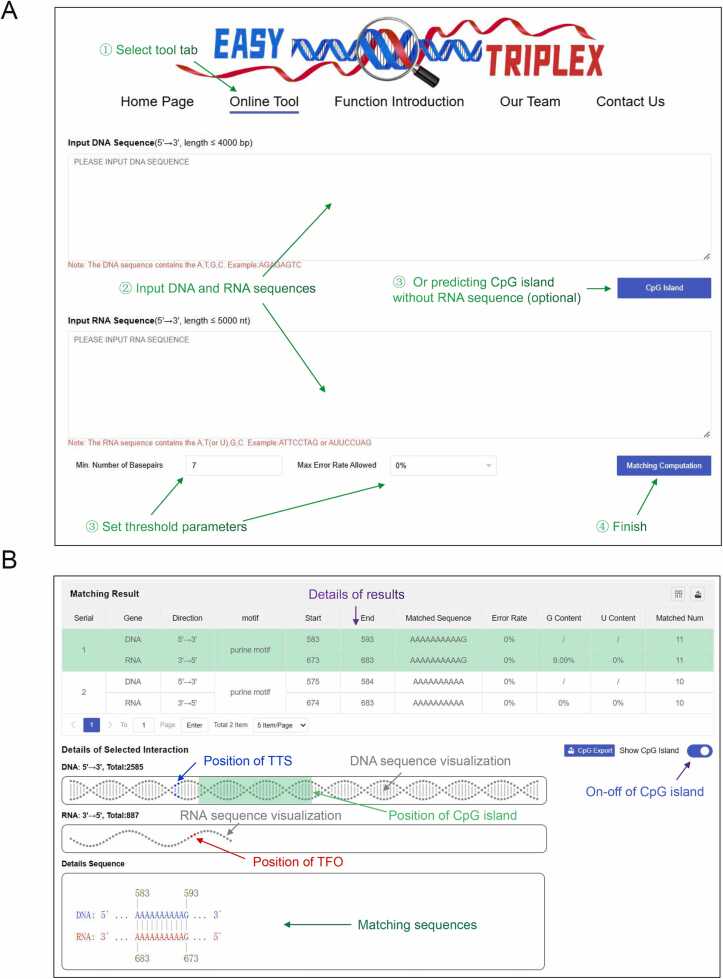
**Example of input data and output results.** (A) Screenshot of the online interface for inputting DNA and RNA sequences. (B) Example screenshot of the output interface displaying the results.

Upon selecting the "Online Tool" tab, users can input the desired DNA and RNA sequences while simultaneously specifying corresponding threshold parameters. If the primary interest is solely in the predicted CpG islands, the user is not required to input the RNA sequence, as this step is optional. For matching prediction, the parameters primarily include the minimum number of consecutive pairs and the maximum allowable error rate. Finally, users click "Matching Computation" to initiate the result computation process (Fig. 5A). In the output display interface, users encounter a list of predicted triplexes. Within each item, the "Matching Result" shows the full length of the DNA and RNA chains in gray, with the blue and red portions representing the respective positions of the TTS and TFO within the DNA and RNA chains, along with their specific positions displayed below. When CpG island computation is enabled, a semitransparent green box will appear on the DNA double helix, indicating the relative position of the CpG island within the DNA double helix and displaying specific information (Fig. 5B). For specific usage instructions, please refer to the downloadable handbook on the website.

### 3.3 Practical Application of Easy Triplex

**Fig. 6:**
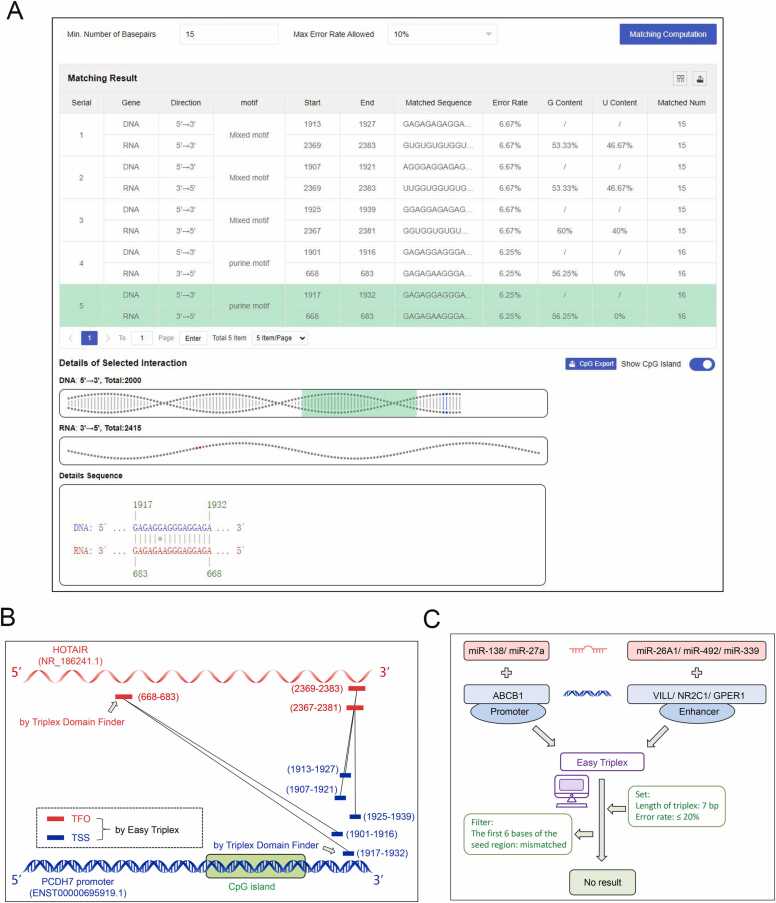
**Practical application of Easy Triplex.** (A) Online screenshot of the binding prediction results between HOTAIR and the PCDH7 promoter sequence. (B) Summary output of the binding prediction between HOTAIR and the PCDH7 promoter sequence utilizing Easy Triplex, including the distributions of the TSS and TFO. The arrows indicate the predicted results from the original study's Triplex Domain Finder, and the relative positions of the blue and red bars correspond to their respective positions on the sequence. (C) The binding prediction process and results using Easy Triplex for 5 known NamiRNAs and their corresponding promoter and enhancer sequences.

To validate the functionality of Easy Triplex, we selected the known lncRNA HOTAIR for predictive analysis. Previous reports indicate that HOTAIR can bind to PCDH7 promoter region and participate in its methylation regulation. It is believed that the mechanism involves the formation of a DNA—RNA triplex structure between HOTAIR and the PCDH7 promoter [16]. Here, we input the sequences of the HOTAIR and PCDH7 promoter into Easy Triplex, with parameters set for 16 consecutive base pair matches and an error rate of ≤ 10 %. The results revealed the presence of multiple TTSs of HOTAIR within the PCDH7 promoter region (Fig. 6A). Upon summarizing the results, we found that Easy Triplex predicted 3 TFOs and the corresponding 5 TTSs. Interestingly, one of the TFOs and matching TTSs predicted using Triplex Domain Finder (applied by the authors) in this publication were also discovered using Easy Triplex (Fig. 6B). This finding indicates that Easy Triplex is capable of achieving precise and comprehensive predictive functionality. Furthermore, when we activated CpG island prediction, we found that these TTSs were all located near CpG islands. This result may further assist the authors in explaining how HOTAIR may influence PCDH7 promoter methylation to regulate its expression.

On the other hand, previous research has revealed potential interactions between miRNAs and target DNA, which can affect gene expression [17]. In vitro experiments have also demonstrated the ability of miRNAs to form triple helix structures with DNA [18]. Some studies have shown that miRNAs interact with target gene promoters; for example, miR-138 and miR-27a may regulate the transcription of ABCB1 by interacting with its promoter [19], [20]. Furthermore, reports have indicated that miR-26A1 and miR-492 directly bind to DNA enhancers as regulatory elements to mediate enhancer activation [21], [22]. These miRNAs, which function in the nucleus, are collectively referred to as nuclear-activating miRNAs (NamiRNAs). When interacting with enhancers, NamiRNAs may also depend on the AGO protein, as observed for miR-339 [23]. However, to date, none of these studies have presented evidence demonstrating the ability of miRNAs to directly interact with DNA through triple helix structure formation, and the studies have not explored specific binding sites of NamiRNAs on DNA. Here, utilizing Easy Triplex, we predicted the binding sites of miR-138 and miR-27a in the ABCB1 promoter, as well as the binding sites of miR-26A1, miR-492, and miR-339 in their respective target gene enhancers. We set the parameters to the lowest standard on the basis of the actual situation of the miRNA, which requires only 7 consecutive base pair matches and an error rate of ≤ 20 %. Additionally, we stipulated that the first 6 base pairs of the miRNA seed region must be consecutively paired as a final filtering criterion. However, the final results indicated that none of the mentioned miRNAs exhibited the potential to form a triplex with the reported target DNA (Fig. 6C). These findings suggest that miRNAs may encounter greater difficulty in completing Hoogsteen pairing.

## 4 Discussion

The formation of triplexes is based on the pairing of DNA and RNA. Although this process inevitably involves the participation of some accessory proteins, the precise local base pairing of RNA as an upstream regulatory molecule is a crucial prerequisite for accurately regulating target genes [24]. Currently, noncoding RNAs are involved primarily in triplex formation, with lncRNAs being the predominant type [25]. Considering the significantly longer length of lncRNAs compared to other non-coding RNAs, Easy Triplex sets higher matching requirements to ensure result verifiability. For the TTS, only G and A are allowed by default, closely following canonical Hoogsteen base-pairing principles to maximize predictive reliability. Additionally, the threshold for parameter settings defaults to ensure at least 7 consecutive pairings, with a maximum error rate ≤ 20 %. By controlling the error rate, this approach aims to select more recommended predictive results while considering the effectiveness of short noncoding RNA binding when predicting such RNA. Therefore, Easy Triplex is not only suitable for predicting the binding of lncRNAs to target DNA but also takes into account the applicability of short-chain RNAs, such as NamiRNAs.

In practical applications, Easy Triplex has demonstrated the ability to predict triplexes effectively for lncRNAs. For the prediction of NamiRNAs and their TFOs, although research has indicated the potential role of NamiRNAs in targeting promoters and enhancers, current evidence, including our own predictions and publications, does not support the direct impact of miRNA on target gene expression through triplex formation. This may be attributed to the short sequence length of miRNA, leading to base randomness that makes it difficult to meet the requirements of Hoogsteen pairing. Additionally, previous studies have suggested that the nuclear activity of miRNAs may require the assistance of AGO proteins, indicating that miRNA—DNA interactions may necessitate strict pairing requirements, particularly in seed sequence pairing [23], [26]. miRNAs may even pair with DNA single strands to form R-loop-like structures, thereby exerting the function of enhancer-associated RNAs [27]. The number of functionally characterized NamiRNAs is currently limited; thus, the full potential of NamiRNAs in triplex formation remains uncertain. Nonetheless, this presents an intriguing research direction, and our tool can potentially assist in future investigations in this area.

Easy Triplex provides users with the flexibility to set parameters as needed, allowing for versatile usage, particularly in conducting in vitro experiments to validate long probes and analyze error rates in special circumstances. Additionally, reports indicate that lncRNA binding to DNA may mediate DNA methylation and thus regulate the expression of target genes [28], [29]. Easy Triplex can directly provide the location and distribution of CpG islands on the DNA sequence during result export. This feature is particularly beneficial for researchers focused on epigenetics and faced with multiple potential TTSs, as it aids in making targeted selections. Previous investigations have also suggested that the percentage of G and U in TFOs may directly affect the stability of the triplex structure [30], but this conclusion remains controversial and requires further confirmation. Therefore, Easy Triplex provides the corresponding percentages of G and U in TFOs during result export, allowing users to consider their own research purposes and parameter settings when selecting potential triplexes.

As the pioneering software for predicting triplex structures, Triplexator defines TFOs and TTSs [31]. Like Easy Triplex, Triplexator also operates on the basis of canonical pairing principles. Triplexator identifies putative triplex interactions between classified TFOs and TTSs by detecting maximal tracts of subsequences that adhere to any of the aforementioned triplex motifs. However, the output results from Triplexator do not impose strict requirements on the length of the triplex or the degree of purine coverage in the TTS. This may lead to relatively high error rates in the predicted triplexes. LongTarget was subsequently introduced as another online computational tool for predicting triplexes [32]. Its primary advantage lies in not being limited to the canonical Hoogsteen pairing rules but rather expanding the DNA—RNA pairing scenarios to 24 cases, with 6 reflecting Hoogsteen pairing and the remaining 18 involving reverse Hoogsteen pairing. The LongTarget algorithm constructs ideal triplex RNA sequences based on the target DNA, resulting in 48 types of RNA sequences. The algorithm then aligns the user-provided sequences with the ideal sequence, generating multiple potential TFOs on the basis of their participation in the ideal sequence, distribution density, and in vitro stability. Although this innovative algorithm and approach may indeed predict more TFOs, the tool has a longer computational time, and the algorithm still requires further optimization. Moreover, the interpretation of the output results from LongTarget can be challenging, especially when predicting short noncoding RNAs and constructing in vitro probes. On this basis, another locally available software called Triplex Domain Finder uses a background set of genomic regions to predict triplexes [33]. This software distinguishes itself by not restricting the prediction to individual TFOs but instead using overlapping TFO sequences as the DNA binding region. This enables the derivation of the total number of potential TTSs in the gene region associated with a single TFO. The total number of TTSs is then compared with the number of predicted triplex interactions between the DNA-binding domain and a representative set of background genomic regions to determine putative triplex-forming domains. Triplex Domain Finder requires installation and has certain platform and operating system constraints. Additionally, the early-stage calculations of this software, particularly the prediction of TFOs, may depend on TRIPLEXES and Triplexator, making overall usage inconvenient. Compared with all the mentioned tools and software, each has its own strengths and limitations due to their different emphases and logical frameworks. Easy Triplex stands out for its flexibility in parameter settings, reasonable default thresholds, and real-time display of the CpG island distribution, making it more practical and user friendly.

A recently launched prediction platform named TRIPBASE, which relies on experimental data and the Triplexator tool to conduct widespread triplex predictions based on human genome data, has emerged [34]. However, owing to its recent launch, its accuracy and practicality still require further validation. This also highlights the fact that Easy Triplex currently does not incorporate genomic information; therefore, it is unable to provide a whole-genome range search. This poses obstacles to the search for and prediction of relevant TTSs for NamiRNAs as mentioned earlier. Additionally, Easy Triplex is based exclusively on 4 canonical Hoogsteen pairing rules and has relatively stringent default condition settings, such as the minimum number of consecutive pairings and the purine coverage of TTS, which may result in the omission of certain potential triplexes during the computation process. Moreover, it is important to acknowledge that Easy Triplex has not yet established an ideal result evaluation system and thus cannot provide corresponding scores and ranking recommendations for output results. These shortcomings will be gradually addressed in future online updates.

The triplex, as the structural basis for RNA—DNA interactions, plays a crucial role in molecular regulation. Owing to the precise sequence pairing, RNA regulation of DNA exhibits specificity and targeting. However, there are still numerous unknowns regarding the regulatory mechanisms involved in these processes, particularly the roles played by short noncoding RNAs. Therefore, Easy Triplex is poised to offer significant practicality and convenience in this field in the future. Furthermore, as we continue to update and upgrade its functionality, its applicability will be optimized in line with advancements in research.

## Funding

This work was by 10.13039/501100001809National Natural Science Foundation of China (Grant numbers: 82472694 and 82103166), 10.13039/501100007129Natural Science Foundation of Shandong Province (Grant numbers: ZR2024MH029), Youth Innovation Technology Project of Higher School in Shandong Province (Grant numbers: 2022KJ267) and Research Start-up Funds of Shandong Second Medical University (No. 04102001 and 02194001).

## CRediT authorship contribution statement

**Zhao Li:** Resources, Methodology, Investigation. **Shi Min:** Methodology, Investigation. **Gong Cheng:** Writing – original draft, Validation. **Li Zhen:** Writing – original draft, Visualization, Validation, Supervision. **Liu Jianming:** Software, Resources, Methodology, Investigation. **Zhu Shunshun:** Investigation, Funding acquisition, Formal analysis. **Liang Fan:** Visualization, Software, Methodology, Investigation. **Luo Hao:** Resources, Methodology, Investigation. **Guo Tao:** Writing – original draft, Supervision, Project administration, Funding acquisition, Data curation, Conceptualization. **Xian Lintao:** Software, Resources, Methodology, Formal analysis, Data curation.

## Declaration of Competing Interest

We declare that we have no financial and personal relationships with other people or organizations that can inappropriately influence our work, there is no professional or other personal interest of any nature or kind in any product, service and/or company that could be construed as influencing the position presented in, or the review of, the manuscript entitled “**Easy Triplex: An Online Tool for Predicting the Formation of DNA—RNA Triple Helices**”.

## Data Availability

This is an online tool, and no original data is generated.
