# Supplementary material for: Easy triplex: An online tool for predicting the formation of DNA—RNA triple helices
Source: Comput Struct Biotechnol J. 2025 Apr 9;27:1550–8. doi: 10.1016/j.csbj.2025.04.008 (PMC12018089; doi:10.1016/j.csbj.2025.04.008)
Supplement: Supplementary file 1 — Supplementary material [file mmc1.pdf]

Supplementary Figures

Figure S1

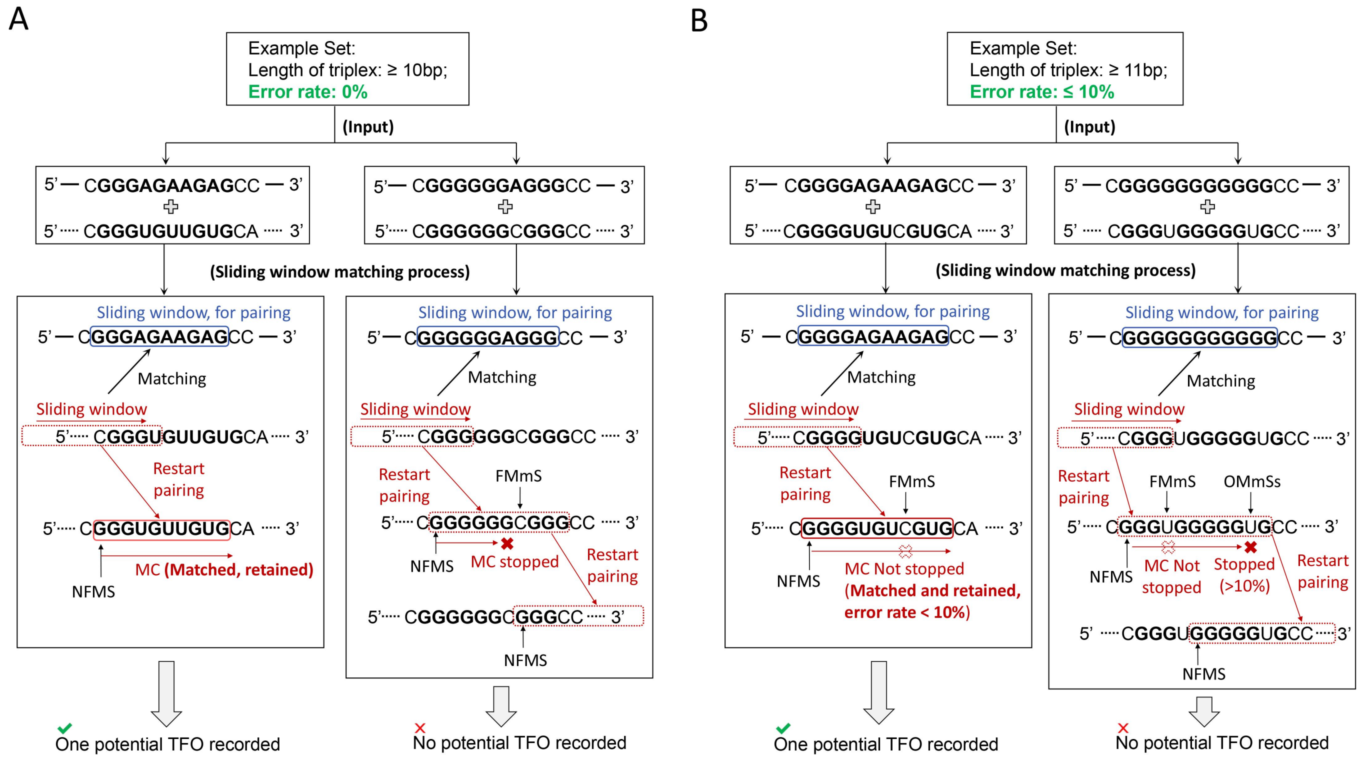

Figure S1. Examples of the sliding window matching process under conditions of zero mismatch rate and non-zero mismatch rate, related to (A) Figure 2B and (B) Figure 2D. All windows are before expansion. MC, matching computation; NFMS, new first matching site; FMmS, first mismatching site; OMmSs, other mismatching sites.

Figure S2

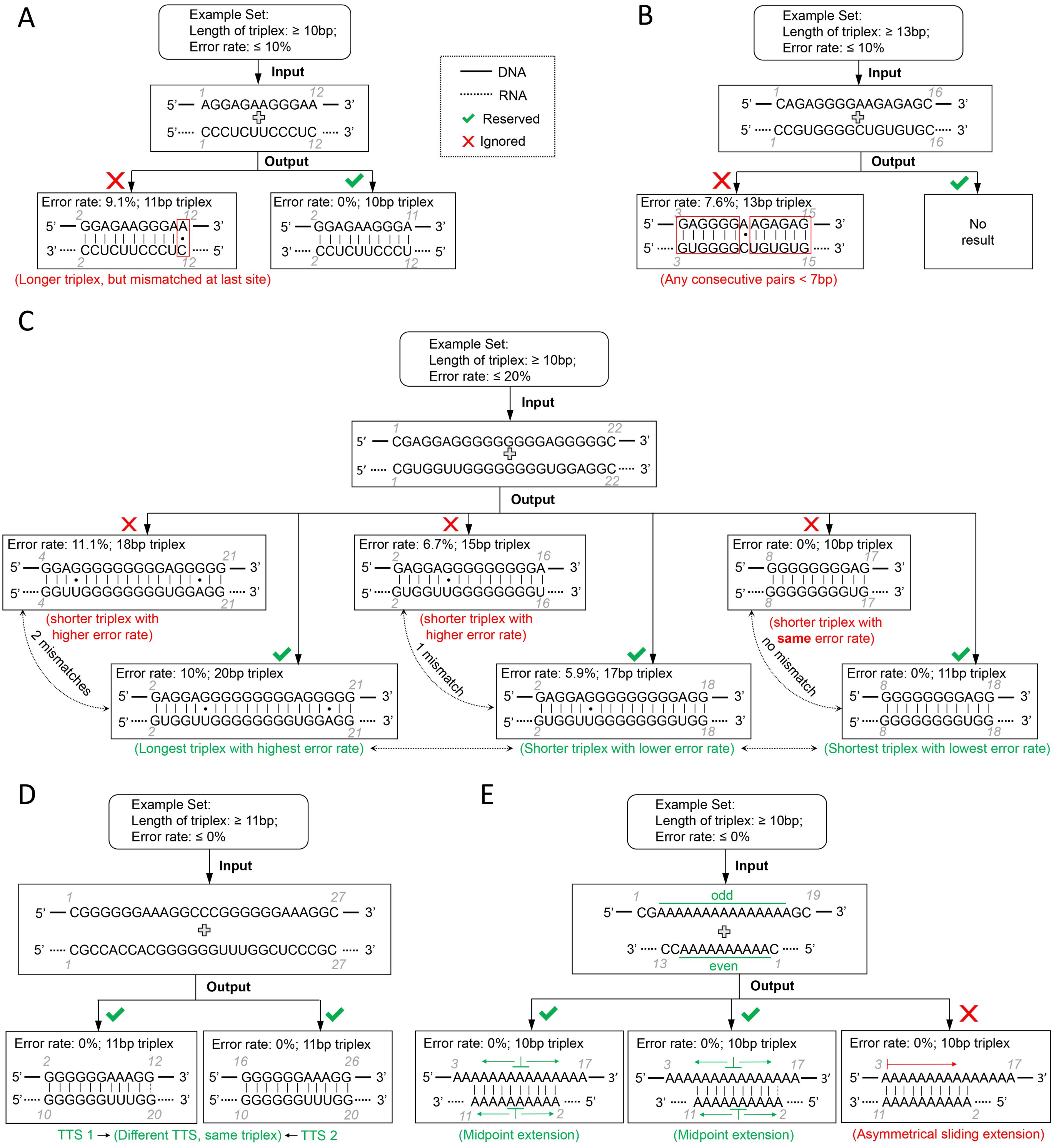

Figure S2. Specific examples of the optimal algorithm related to (A) Figure 3B, (B) Figure 3C, (C) Figure 3D, (D) Figure 3E, and (E) Figure 3F.
